# MethylPurify: tumor purity deconvolution and differential methylation detection from single tumor DNA methylomes

**DOI:** 10.1186/s13059-014-0419-x

**Published:** 2014-08-07

**Authors:** Xiaoqi Zheng, Qian Zhao, Hua-Jun Wu, Wei Li, Haiyun Wang, Clifford A Meyer, Qian Alvin Qin, Han Xu, Chongzhi Zang, Peng Jiang, Fuqiang Li, Yong Hou, Jianxing He, Jun Wang, Jun Wang, Peng Zhang, Yong Zhang, Xiaole Shirley Liu

**Affiliations:** Department of Mathematics, Shanghai Normal University, Shanghai, China; Department of Biostatistics and Computational Biology, Dana-Farber Cancer Institute and Harvard School of Public Health, Boston, Massachusetts USA; Department of Bioinformatics, School of Life Science and Technology, Tongji University, Shanghai, China; Tongji University Advanced Institute, Translational Medicine, Shanghai, China; Center for Functional Cancer Epigenetics, Dana-Farber Cancer Institute, Boston, Massachusetts USA; BGI-Shenzhen, Shenzhen, China; The First Affiliated Hospital of Guangzhou Medical College, Guangzhou, China; Department of Biology, University of Copenhagen, Ole Maaløes Vej 5, 2200 Copenhagen, Denmark; Princess Al Jawhara Center of Excellence in the Research of Hereditary Disorders, King Abdulaziz University, Jeddah, 21589 Saudi Arabia; Macau University of Science and Technology, Avenida Wai long, Taipa, Macau, 999078 China; Department of Medicine, University of Hong Kong, Pokfulam, Hong Kong; Department of Thoracic Surgery, Shanghai Pulmonary Hospital of Tongji University School of Medicine, Shanghai, China

## Abstract

We propose a statistical algorithm MethylPurify that uses regions with bisulfite reads showing discordant methylation levels to infer tumor purity from tumor samples alone. MethylPurify can identify differentially methylated regions (DMRs) from individual tumor methylome samples, without genomic variation information or prior knowledge from other datasets. In simulations with mixed bisulfite reads from cancer and normal cell lines, MethylPurify correctly inferred tumor purity and identified over 96% of the DMRs. From patient data, MethylPurify gave satisfactory DMR calls from tumor methylome samples alone, and revealed potential missed DMRs by tumor to normal comparison due to tumor heterogeneity.

**Electronic supplementary material:**

The online version of this article (doi:10.1186/s13059-014-0419-x) contains supplementary material, which is available to authorized users.

## Background

DNA methylation is an important epigenetic mark controlling gene expression, thus playing pivotal roles in many cellular processes including embryonic development [1], genomic imprinting [2,3], X-chromosome inactivation [4], transposable element repression [5], and preservation of chromosome stability [6]. Aberrant DNA methylations are known to be associated with human diseases such as cancers, lupus, muscular dystrophy, and imprinting-related birth defects [7-14]. Whole genome bisulfite sequencing (WGBS) and reduced representation bisulfite sequencing (RRBS) [15-18] are popular techniques to profile genome-wide methylation at a nucleotide resolution [19]. The sodium bisulfite treatment in these techniques converts the unmethylated cytosines to uracils, while leaving the methylated cytosines unchanged. Mapping the bisulfite-treated DNA sequences to the genome not only gives precise location but also the quantitative levels of DNA methylation. In recent years, WGBS and RRBS have been increasingly used to profile the DNA methylation patterns between tumors and their normal counterparts, where differential methylated regions not only serve as important cancer biomarkers and therapeutic targets, but also provide insights to the mechanism of tumorigenesis and progression [20-22].

Despite the popularity of WGBS and RRBS, these techniques suffer from the following practical limitations in cancer research. First, differential methylation analysis is conducted as cancer to normal comparisons, requiring additional resources to collect, process, sequence and analyze the normal tissues adjacent to the cancer tissues. Second, in most cases, tumor tissues are not pure but contain unknown quantities of normal cells [23]. As a result, the contamination of normal cells in the tumor sample complicates the differential methylation calling between tumor and normal. Some pioneering works estimated tumor purity based on gene expression or SNP array data [23-27], but to the best of our knowledge, there have been no reported algorithms estimating tumor purity from WGBS or RRBS data. One approach used in previous expression-based studies is to train the algorithm on a large number of datasets from tumor or normal cells [28] or on expression signatures generated from such large data cohorts [29]. However, the expression observed from the cohorts may not best recapitulate a specific tumor sample, thus could give biased estimates. Another approach is to see whether regions with known germline variants or somatic mutations have differential expression or methylation on the different alleles [30]. This approach is limited in the number of regions it can investigate, thus could not identify or resolve differential regions that do not contain sequence variations [9,31-33].

We propose a statistical approach called ‘MethylPurify’ to estimate tumor purity and identify differentially methylated regions from DNA methylome data on tumor samples alone, without any prior knowledge from other datasets. MethylPurify assumes that, in pure cell populations, methylation levels of bisulfite-sequencing reads are consistent within short genomic intervals except in a small number of regions with allele-specific methylation (ASM). This phenomenon has been reported in several studies by examining the co-methylation states of adjacent CpGs within a region especially for CpG Islands [34-36]. Inconsistent methylation on the CpGs within a single read might be due to incomplete conversion of bisulfite treatment. Even though tumors are often heterogeneous, most tumors follow clonality [37-40], meaning the initiation and continued growth of a tumor is usually dependent on a single population of tumor cells. The small population of heterogeneous tumor cells often does not interfere with differential methylation detection, and this assumption has also been used for differential methylation studies by paired tumor to normal comparison. In samples with two cell population components such as tumor and normal, there will be a large number of regions differentially methylated between the two components where bisulfite reads show discordant methylation levels. Since most tumor samples have normal contamination, MethylPurify examines all the regions in the genome with reads showing discordant methylation levels and estimates the mixing ratio of the two components. With the mixing ratio estimate, MethylPurify examines each such regions, assigns reads to the two components, and infers the methylation level of each component. We evaluated the performance of MethylPurify on simulations mixing bisulfite reads from two human breast cell lines at different ratios and on real lung adenocarcinoma tissues where the data from adjacent normal tissue were available but withheld from the algorithm. In each case, MethylPurify gave satisfactory performance in estimating the tumor purity and in identifying differential methylation regions between the components.

## Results and discussion

### Computational framework

**Figure 1:**
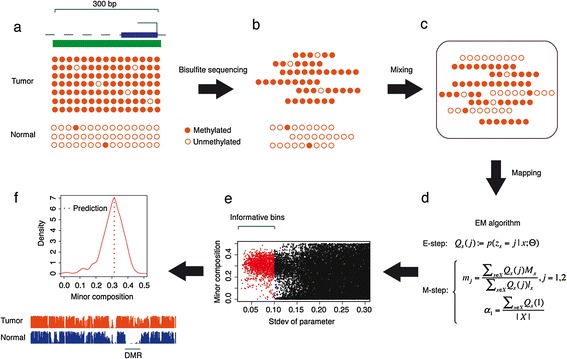
**Overview of MethylPurify. (a)** A differentially methylated region (DMR) between tumor and normal cells. Solid and hollow red circles represent methylated and unmethylated cytosines, respectively. **(b)** Short reads from two cell populations after bisulfite treatment and sonication. **(c)** A library of bisulfite reads in a mixture of two cell populations. **(d)** EM algorithm iteratively estimates three parameters: the minor composition (α_1_) and the methylation level of each population (m_1_, m_2_) in M step, and assigns reads to each population in E step. **(e)** Among all 300 bp bins, the parameters estimated from informative bins converge on a final mixing ratio estimate. **(f)** Top, density plot of predicted minor component from selected informative bins. Bottom, separated methylation level of tumor and normal cells based on the predicted mixing ratio, and DMRs are detected as consecutive differentially methylated bins (DMBs).

The conceptual framework of MethylPurify is shown in Figure 1. Under the assumption that tumor tissues often contain two major components of cells, that is, tumor and normal, MethylPurify only takes WGBS or RRBS data from a tumor tissue as input, and tries to infer the unknown fraction of normal cells within. After removing duplicated reads and mapping them with BS-map [41], MethylPurify divides the reference genome into small 300 bp bins and assigns reads mapped to each bin. The true methylation levels in most bins are similar between the two components and thus not informative to tumor purity inference or differential methylation analysis. Instead, MethylPurify aims to find informative bins that have differential methylation between normal and tumor cells, and use them to help infer the tumor purity and the methylation level of each component. It relies on the following characteristics of the DNA methylome data: (1) all CpG cytosines within a short genomic interval (approximately 300 bp) in a pure cell population share similar methylation levels which are either mostly methylated or mostly unmethylated [36]; (2) the number of bisulfite reads mapped to each genomic interval to tumor and normal cells are in accordance with their relative compositions in the mixture, subject to standard sampling noise.

MethylPurify uses the following mixture model to estimate the two components in the tumor methylome data. Given a mixture of bisulfite reads from two components, the relative compositions of the minor and major components can be represented as *α*_1_ and 1 - *α*_1_, and the methylation levels of the two components within each 300 bp bin can be represented as *m*_1_ and *m*_2_, respectively. Given initial parameter values of *α*_1_, *m*_1_, and *m*_2_, each read in a bin can be assigned to its most likely component; given the read assignment in a bin, parameter values of *α*_1_, *m*_1_, and *m*_2_ can be re-estimated to maximize the probability of seeing the specified read assignment. For each 300 bp bin across the genome, MethylPurify uses expectation maximization (EM) to iteratively estimate parameters and assign reads until convergence (see Methods section for details).

Due to the sampling noise and other confounding biases, *α*_1_ estimates from individual bins will be distributed around the true value. To reach a more reliable mixing ratio from all *α*_1_ estimates, MethylPurify uses the following bootstrapping approach to prioritize the informative bins. First, it selects only bins with over 10 CpGs, 10-fold read coverage (termed qualifying bins thereafter), then samples equal number of reads as the actual number of reads in each bin with replacement 50 times to get 50 sets of EM converged *α*_1_, *m*_1_, and *m*_2_ parameters. To avoid complications of copy number aberrations (CNA) in cancer at this step, MethylPurify filters bins in regions with frequent copy number alterations as well as their 1,000 bp flanking regions, and only selects one qualifying bin within each CpG island. Then MethylPurify finds the 500 bins with the smallest parameter variance in the 50 sampling and uses the mode of their *α*_1_ estimate as the *α*_1_ for the whole tumor sample (Figure 1e,f). With the sample *α*_1_, a few EM iterations in each bin could quickly converge on the *m*_1_ and *m*_2_ estimates and read assignment across the genome. To avoid local maxima of EM, MethylPurify starts from two distinct initial values of *m*_1_ and *m*_2_ in each bin, representing *α*_1_ component being hyper- and hypo-methylated, and the convergence point with higher likelihood is selected as the final prediction (see Methods section for details).

The output of MethylPurify will report the mixing ratio of the two components (*α*_1_: 1 - *α*_1_) in the whole sample and the methylation level of each component (*m*_1_ and *m*_2_) in each qualifying bin across the genome. MethylPurify could also detect differentially methylated regions (DMRs) as consecutive differentially methylated bins (DMBs).

### Inference of mixing ratio from simulated mixture of bisulfite reads from tumor and normal cell lines

To validate MethylPurify in estimating the mixing ratio, we used simulated mixture of whole genome bisulfite sequencing data from two separate breast cell lines [22]. HCC1954 cell line (thereafter refer to as HCC) is derived from an estrogen receptor (ER)/progesterone receptor (PR) negative and ERBB2 positive breast tumor, and human mammary epithelial cell line (HMEC) is immortalized from normal breast epithelial cells. Bisulfite sequencing for the two cell lines have slightly different read lengths (approximately 70 to 100 bp) and sequencing coverage (27-fold and 20-fold, respectively). We randomly sampled bisulfite reads from the two cell lines at 20-fold total coverage with varying mixing ratios from 0:1 (all HMEC) to 1:0 (all HCC) with a step of 0.05.

**Figure 2:**
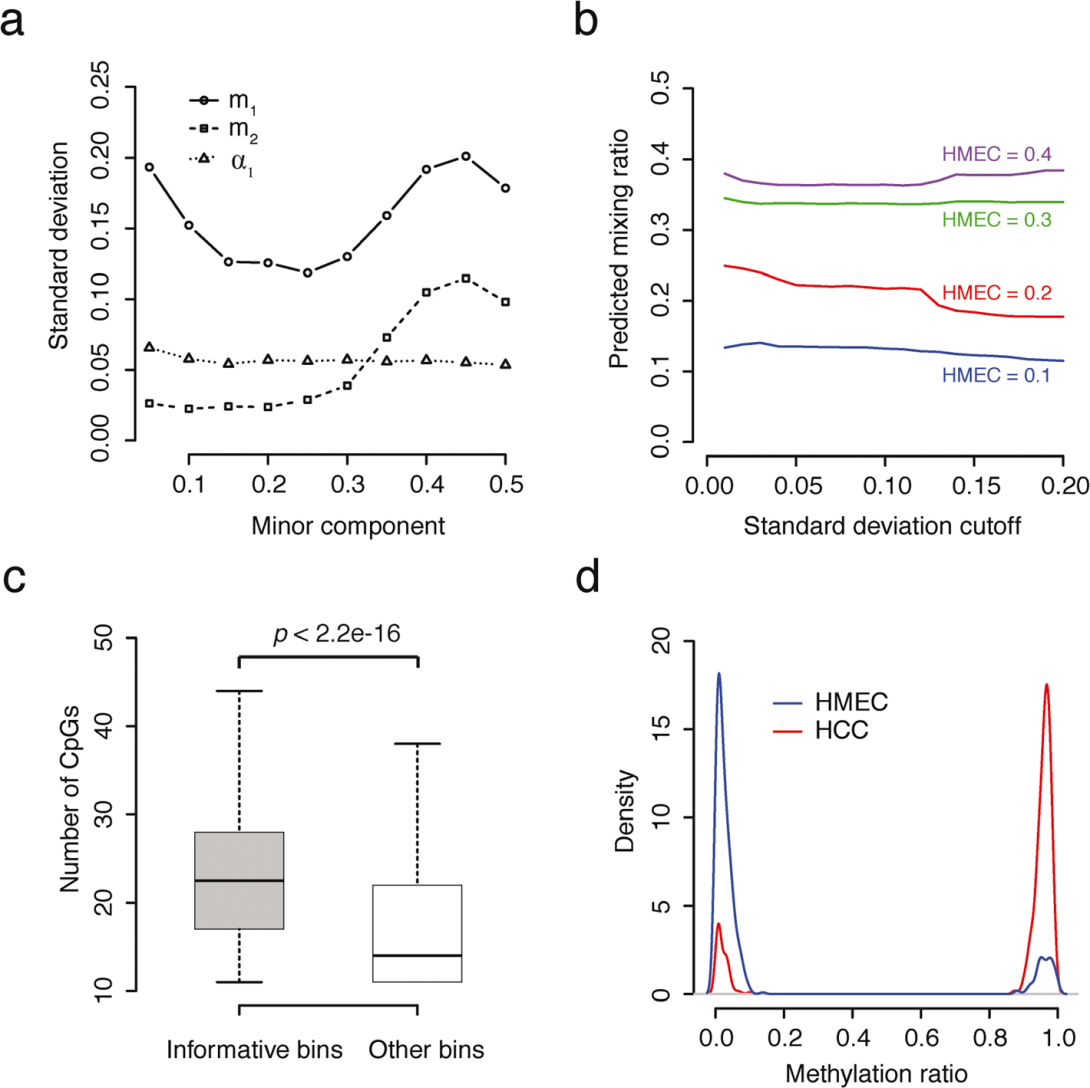
**Parameter estimation and properties of informative bins. (a)** Averaged standard deviations of three free parameters at different mixing ratios by bootstrap sampling of HCC (breast cancer) and HMEC (normal mammary epithelial) cell lines for all qualifying bins. **(b)** Predicted mixing ratios at different standard derivation cutoffs of the minor composition. **(c, d)** Some properties of informative bins compared with genome-wide background. **(c)** The number of CpG counts in informative bins *vs.* the rest bins with over 10 fold read coverage. **(d)** Distribution of predicted methylation levels of informative bins in each cell line.

We first examined how the parameter estimation varies with changing inputs. At different mixing ratios, the average variance (of all qualifying bins by bootstrapping) of the minor component percentage *α*_1_ is very small and stable (Figure 2a). The variance of *α*_1_ initially increases with the mean of *α*_1_, but is suppressed as *α*_1_ approaches 0.5 since *α*_1_ is designated as the minor component to be always ≤0.5 in our model. In contrast, the estimated methylation level of the minor component *m*_1_ is the most variable. This is reasonable because at low *α*_1_ (close to 0), the minor component has very little read coverage; at high *α*_1_ (close to 0.5), it is sometimes difficult to determine which component is minor so *m*_1_ could fluctuate depending on whether MethylPurify assigns the methylated or unmethylated reads to the minor component.

Since *m*_1_ is the most variable among the three parameters and dominates the sum of the variances, MethylPurify later only uses the standard deviation (stdev) of *m*_1_ from bootstrapping to rank all qualifying bins. Indeed, the informative bins, defined as qualifying bins with *m*_1_ stdev <0.1 (after filtering CNA regions and selecting one bin with smallest stdev from each CpG island), in general give very stable *α*_1_ estimates at different mixing ratios (Figure 2b). A closer examination of the informative bins found that they often contain significantly more CpGs (Figure 2c), and have a strong dichotomy of reads being either mostly methylated (1) or mostly unmethylated (0) (Figure 2d). So in the remaining text, the top 500 informative bins with the smallest parameter variance by bootstrap were used to vote for the mixing ratio for the whole sample.

**Figure 3:**
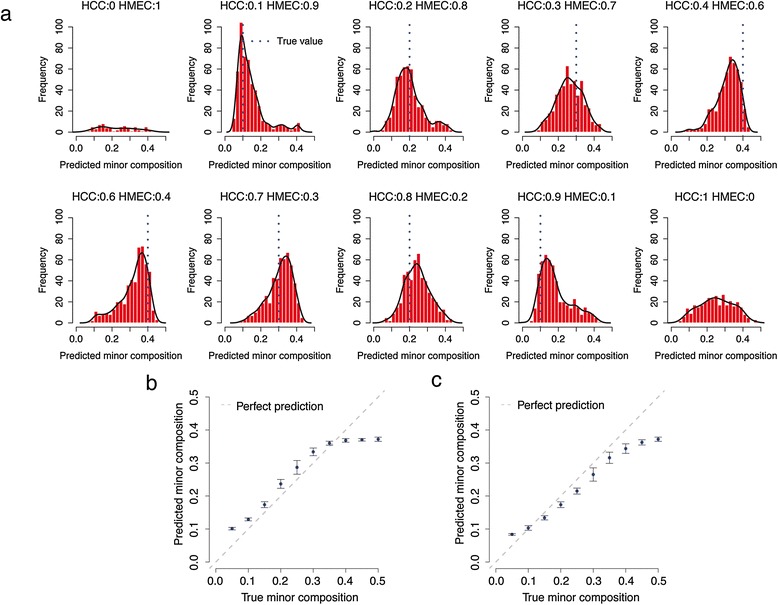
**Prediction performance on simulated data. (a)** Histogram of the predicted mixing ratio from selected informative bins insimulation results when bisulfite reads of HCC and HMEC cell lines are mixed at different ratios. Dotted blue lines highlight the true minor components. Black line is the density of the predicted mixing ratio. **(b, c)** Predicted minor compositions (α_1_) at different mixing ratios where the composition of tumor cell line is above 50% **(b)** or below 50% **(c)**. Error bars represent standard derivations derived from 20 mixing simulations.

We then evaluated whether MethylPurify could correctly infer the mixing ratio of the two components. When given a pure cell line without mixing, MethylPurify correctly reported a warning for insufficient number (66 and 322 for HMEC and HCC cell lines, respectively) of informative bins. Further examination of such bins in HCC cell line suggested that they have significant overlap with ASM regions [22] (*P* = 0.0086 by Fisher’s exact test). For all samples with real mixing, MethylPurify identified sufficient number of informative bins across the genome (see Additional file 1: Figure S1 as an example), and their respective *α*_1_ estimates are often centered around the true *α*_1_ (Figure 3a). Over 20 repeated simulations at each mixing ratio, MethylPurify gives predicted *α*_1_ that tightly surrounds the true *α*_1_ with two interesting twists (Figure 3b,c). The first is that since MethylPurify dictates *α*_1_ to represent the minor component, *α*_1_ estimates tend to be slightly lower when the mixing is close to 0.5:0.5. The second is that MethylPurify tends to slightly under estimate the cancer component. This might be because even as cell lines, the cancer HCC is more heterogeneous than the normal HMEC, as supported by the larger number of informative bins in HCC than HMEC alone, causing the EM algorithm to assign a small portion of the HCC reads to the HMEC component. This implies that in tumor samples, MethylPurify might also tend to slightly underestimate the tumor percentage due to tumor heterogeneity.

### Detection of differentially methylated bins in the simulated mixture

**Figure 4:**
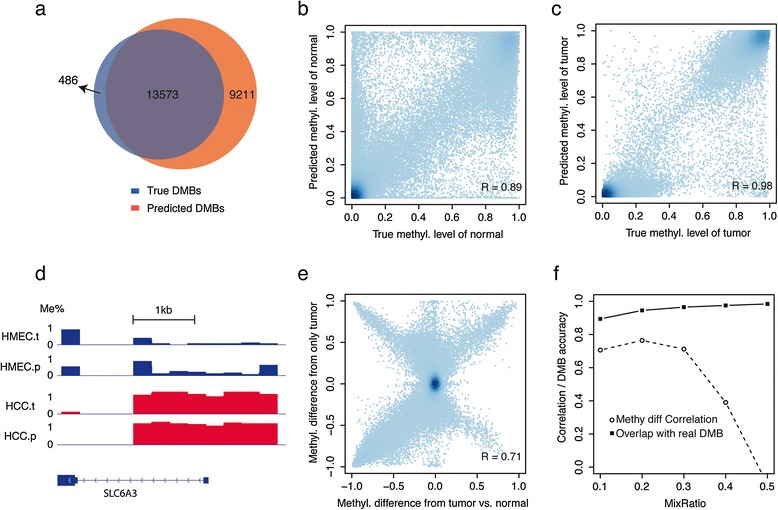
**Predicted DMBs (DMBs predicted to have methylation difference over 0.5 from mixture reads by MethyIPurify) are compared with true DMBs (DMBs inferred from BS-seq reads in two separate cell lines) in HCC and HMEC cell lines. (a)** Overlap between predicted (from mixture, red) and true (blue) DMBs in the mixture of HCC1954 (70%) and HMEC (30%). **(b, c)** Correlations of predicted and true methylation levels in normal **(b)** and tumor cell line **(c)** for qualifying bins. **(d)** An example of differentially methylated region between HCC and HMEC cell lines. Each point represents one qualifying bin of length 300 bp. HMEC.t and HCC.t are true methylation profiles in this region, while HMEC.p and HCC.p are predicted methylation profiles from mixture reads. **(e)** Correlation of predicted and true methylation differences. **(f)** DMB prediction sensitivity and correlation of methylation between predicted and true differential methylation at different mixing ratio of the two cell lines.

We next evaluated whether MethylPurify could correctly predict the methylation level of each component in the mixture and identify the differentially methylated regions between the two components. At HCC and HMEC mixing ratio of 0.7:0.3, we analyzed all 90,748 qualifying bins (300 bp with over 10 CpGs and over 10-fold coverage) to evaluate the performance. Under the gold standard of methylation difference >0.5 between the two pure cell lines, we found that MethylPurify could predict differentially methylated bins at 96.5% sensitivity and 88.0% specificity (Figure 4a). At coverage range from 10-fold to 40-fold, the performance of MethylPurify decreases only slightly with decreased coverage, although the number of qualifying bins with enough coverage decreases (Additional file 2: Figure S2).

Detailed examinations revealed that in the true positive predicted regions HMEC is often fully unmethylated (0) while HCC is fully methylated (1). This is consistent with many studies showing that cancer samples often have global hypomethylation and CpG promoter hypermethylation [42-45]. In contrast, in the false positive bins, the methylation levels in the individual cell lines are often at intermediate levels (Additional file 3: Figure S3). These might represent the methylation variability regions previously reported in tumor DNA methylation studies [14,46,47], and might cause reads to be assigned to the wrong component. For example, a tumor sample has 1/3 normal and 2/3 cancer, and in one region the methylation level of the normal and cancer components are 0% and 50%, respectively. Assume MethylPurify correctly estimated the minor component *α*_1_ to be 1/3, it would naturally assign the 1/3 methylated reads to the minor normal component, and 2/3 unmethylated reads to the major cancer component. In this case, although MethylPurify incorrectly called the cancer component as hypomethylated, it nonetheless correctly identified this region as differentially methylated, whereas a standard cancer/normal differential call might miss it.

To reduce the above effect of tumor heterogeneity, we removed bins that show strong read methylation variability (var >0.1) in the HCC (Additional file 4: Figure S4). We then examined whether DNA methylation levels of the two components can be correctly estimated in the remaining qualifying bins. The correlation between the true and predicted methylation level is at 0.89 for the minor normal component and 0.98 for the major tumor component, respectively (Figure 4b,c). Figure 4d is an example showing the true and predicted methylation levels from each cell line in the mixture. The predicted methylation difference from the cell line mixture is highly correlated with the estimated methylation difference by directly comparing the two individual cell lines (Figure 4e). Further examination of bins that were called in the wrong directions found many to have lower sequence coverage. This suggests that the mixture sampling might introduce biases, i.e. the mixing at specific bins could be off from the genome-wide ratio of 0.7:0.3. In fact, if we examine only bins with >15-fold coverage, the correlation of methylation difference estimated from individual cell lines *vs.* mixture increased from 0.71 to 0.75.

We then tested the performance of MethylPurify when the normal (HMEC) component of the mixture varies from 0.1 to 0.5. When the mixing ratio is close to 0.5:0.5, determining which component is hypo- or hyper-methylated becomes an unidentifiable problem, so the correlation between the true and predicted methylation difference in the two components drops. Nonetheless, our ability to correctly call regions of differential methylation increases with the minor component percentage, from 89.4% at 0.1:0.9 mixing to 98.4% at 0.5:0.5 mixing, because there is enough coverage on each component to confidently identify bins with discordant methylation reads (Figure 4f).

### Application of MethylPurify to lung cancer tissues

**Figure 5:**
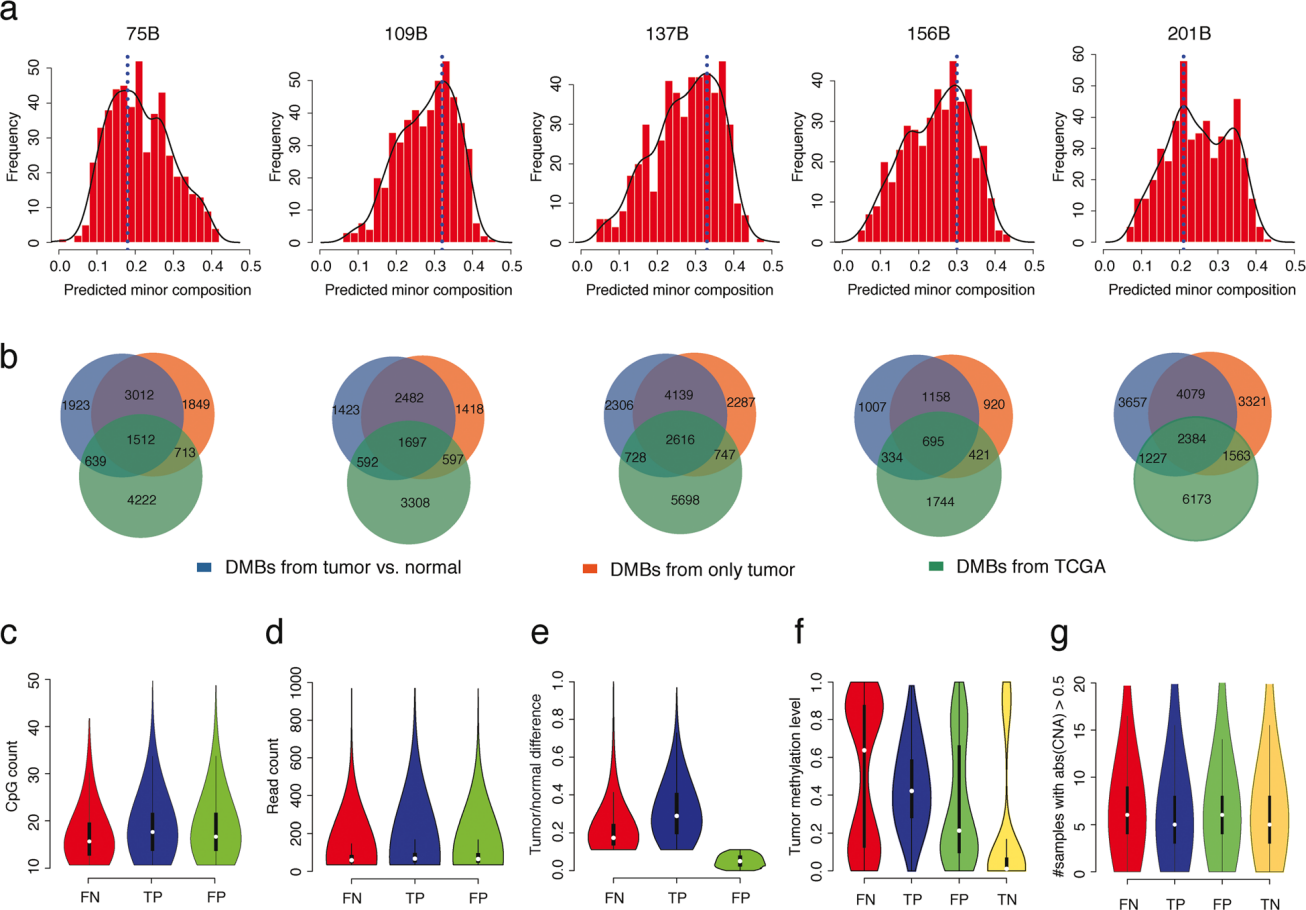
**Application of MethylPurify to the lung adenocarcinoma samples. (a)** Distribution of informative bins and the calculated minor components of five primary lung adenocarcinoma samples. **(b)** The numbers of DMBs inferred from normal-tumor comparison (blue, true DMB), predicted DMB by MethylPurify from only tumor tissue (red) and DMB inferred from TCGA (green) for each sample. **(c-g)** Violin plots show the distributions of CpG counts **(c)**, read counts **(d)**, tumor/normal methylation differences **(e)**, tumor methylation levels **(f)**, and numbers of lung adenocarcinoma samples with copy number alteration in TCGA **(g)** for false negative (FN), true positive (TP), false positive (FP), and true negative (TN) bins.

With the success of MethylPurify on cell line mixing simulations, we next tested MethylPurify on real tumors. We conducted reduced representation bisulfite sequencing on five primary lung adenocarcinoma samples as well as their respective adjacent normal tissues, and obtained approximately 15 to 40 million 90 bp reads for each sample. MethylPurify was able to process each tumor sample within 1 h on a single core, and estimated the normal component in the tumors to be between 18% and 33% (Figure 5a). In these samples, the true normal percentage in each tumor sample is unknown. In addition, methylation differences have been reported to well precede pathological differences, which have been used to predict cancer risk [48]. Therefore, we instead focused on evaluating differentially methylated regions called by MethylPurify from tumor samples alone, using the tumor to normal comparison as the gold standard. In this standard, a 300 bp bin is defined as differentially methylated if the average methylation difference between cancer and normal in the region exceeds 0.5. We also tried other cutoffs to call differential methylation and got similar results (data not shown).

For each sample, we divided the genome into 300 bp bins and only considered qualifying bins with >10 CpG and ≥10-fold read coverage. Due to different sequencing depth on the different samples, the number of qualifying bins in different samples varies. We then examined the Cancer Genome Atlas (TCGA) lung adenocarcinoma methylation data [49] and used the differential methylated regions in TCGA that overlap with the qualifying bins in each sample to determine the number of differential DNA methylated bins to call in each sample. Differentially methylated bins called either from tumor samples alone or from the tumor to normal comparisons are both ranked by their absolute differential methylation levels, respectively. Using the tumor to normal comparison as gold standard, MethylPurify calls in the tumor samples alone could achieve sensitivity of over 57% and specificity of over 91% in the five samples tested (Figure 5b).

We then examined the false negatives and false positive predictions MethylPurify made on the tumor samples alone. Using sample 137B as an example since it has the best sequencing coverage, we found that the regions with false negative predictions often have fewer CpG count (*P* <2.2e-16, t-test, Figure 5c), lower coverage (*P* = 0.0037, Figure 5d), and smaller methylation differences (*P* <2.2e-16, Figure 5e) between tumor and normal. In contrast, the false positive bins are more similar to true positive ones in CpG count (*P* = 0.73) and read coverage (*P* = 0.83). Interestingly, their absolute DNA methylation in the tumor samples show more intermediate levels instead of the dichotomy of 0 for unmethylated or 1 for methylated (Figure 5f), and they often contain many reads with discordant methylation levels. They suggest that such regions are indeed differentially methylated, but were not detected in the normal cancer comparison because tumor heterogeneity reduced the observed normal to tumor methylation difference (Figure 5e). Indeed, among the false positive bins MethylPurify called from tumor alone, 25% to 32% have differential methylation support in TCGA lung adenocarcinoma data (Figure 5b). This suggests that these ‘false positives’ should have been correct calls, but were missed by the tumor/normal comparison potentially due to tumor heterogeneity. This percentage is similar to the 24% to 29% true negative calls with TCGA support, implying that the differential methylation called by MethylPurify from the tumor samples alone is as good as the tumor/normal comparison.

## Conclusion

Tumor impurity has been a challenging technical issue in most cancer molecular profiling projects. Here we present MethylPurify, a statistical method to automatically estimate the purity of tumor samples and to call methylation levels in genome-wide scale for each component based on bisulfite sequencing data. This is the first method of its kinds without the need to train the parameters on many normal, tumor, or cell line data, or can only detect methylation differences at regions with sequence variations from a single sample. In contrast, MethylPurify finds regions with significant number of reads with discordant methylation levels, which are rare in pure cell populations but far more prevalent in tumors with impurities. MethylPurify is able to identify differentially methylated regions from tumor samples alone, thus saving the time and efforts for normal sample processing. The method is especially useful for studies such as glioblastoma, where the normal brain tissues are hard to obtain.

Despite the aforementioned advantages, MethylPurify has some technical limitations. First, MethylPurify depends on having sufficient bisulfite sequencing coverage, preferably at 20-fold or higher, although it will still work at lower coverage if the minority component is reasonably abundant. MethylPurify also relies on short regions containing mostly methylated and mostly unmethylated reads, thus the regions with higher CpG density are more likely to be informative in the estimation of mixing ratio and in identifying differentially methylated regions. In order to detect differentially methylated regions in low CpG regions, MethylPurify requires higher bisulfite sequencing depth. We hope our future work can improve MethylPurify to overcome these limitations.

MethylPurify currently only works for the mixture of two components, and contaminations that consist less than 5% of the sample usually do not interfere with the algorithm prediction. When the sample is very pure such as a cell line, although there might not be enough informative bins, MethylPurify could instead predict ASM. In fact, detecting ASM in pure cell population is a simplified form of MethylPurify. In tumors with impurities, these ASM regions might slightly bias the mixing inference, which was demonstrated in Additional file 5: Figure S5. This sample has 40% normal and 60% tumor, and a region with ASM in normal and loss of ASM and fully methylated in tumor will look like a normal: tumor mixing ratio of 1:4 (Additional file 5: Figure S5b,c). However, since the number of DMRs in tumors are often much larger than the number of ASM regions, this small bias would not affect the mixing ratio inference. The mixture model of MethylPurify can be extended to handle samples with more components. For such cases, deeper sequencing depth and a more sophisticated algorithm are required to automatically determine the number of components in the mixture.

In tumor samples, the normal contamination could be either the minor or major component. Since MethylPurify only infers the ratio of the minor component, the user will need to use pathology information to check whether this is the tumor or normal. Alternatively, examination of a few well-known differentially expressed genes or differentially methylated regions between normal and cancer can also resolve the problem. When the mixing ratio is close to 0.5:0.5, MethylPurify can still identify differentially methylated regions, but will lose the genome-wide phasing and fail to determine whether the regions are hypermethylated or hypomethylated in tumor. This can be improved by examining multiple tumor samples of the same cancer type, which are likely to have different mixing ratios and share many differentially methylated regions.

For metastasized tumors, the normal contamination is from the metastasized site rather than the origin tissue of the tumor, which might result in different differential methylation calls. However, obtaining both metastasized tumor as well as the normal tissue of the tumor origin in practice is quite difficult, so obtaining DMR information directly from the metastasized tumor is still attractive, despite being imperfect. In fact, our test on the lung cancer samples metastasized to the brain (Additional file 6: Figure S6) still identified many of the correct DMRs.

Differentially methylated alleles in SNP regions in the genome have been effectively used to infer ASM. MethylPurify does not rely on genetic variation information, so could detect more ASM or differentially methylated regions that lack genome variations. Since genome variations provide additional layers of information for methylation level and mixing ratio inference [50], it is a good feature to be incorporated in future versions of MethylPurify. In addition to point mutations, somatic copy number aberration (CNA) is also common in cancers, and this also affect tumor/normal methylome comparisons. In fact, some of the false positive (*P* = 0.023) and false negative (*P* = 1.1e-05) predictions from MethylPurify might be due to potential copy number amplifications in the tumors (Figure 5g). However, for bisulfite-sequencing data with sufficient coverage, large regions of CNA might be directly identified from sequence coverage. Therefore, future versions of MethylPurify could estimate their effect in the model to eliminate false positives or false negatives.

## Methods

### Notation and model construction

Suppose a tumor tissue consists of tumor and normal cells. Since the composition of each cell type is unknown, we use the terms ‘major component’ and ‘minor component’ to represent the respective cell types that make up the majority and minority of the cell population in the tumor. In most cases, the tumor cells are the major component. Denote the proportion of minor and major components in a tumor mixture as *α*_1_ and *α*_2_, and their corresponding CpG methylation ratio in a given genomic interval *I* as *m*_1_ and *m*_2_, respectively. The methylation pattern in this interval could be modeled by three free parameters *Θ* = (*m*_1_, *m*_2_, *α*_1_) since *α*_1_ + *α*_2_ = 1. We focus only on methylation patterns at the CpG dinucleotide, since non-CpG tri-nucleotide methylation patterns (mCHG and mCHH, where H = A, C, or T) are shown to be different from CpG methylation [51,52]. Let *X* be a set of bisulfite reads mapped into *I* and *x* be a sequence from *X*. If *x* includes *l*_*x*_ CpG cytosines, then *x* could be represented as a binary sequence $={x}_1{x}_2\cdots {x}_{l_x}$:

$$ {x}_i\kern0.5em =\kern0.5em \left\{\begin{array}{l}1,\kern0.5em \mathrm{if}\ \mathrm{the}\ \mathrm{ith}\ \mathrm{CpG}\ \mathrm{in}\ \mathrm{x}\ \mathrm{is}\ \mathrm{methylated};\\ {}0,\kern0.5em \mathrm{otherwise},\end{array}\right. $$

where *i* = 1, 2, …, *l*_*x*_. Denote ${M}_x={\displaystyle \sum_{i=1}^{l_x}}{x}_i$ as the number of methylated CpG cytosines in *x*, and ${U}_x={\displaystyle \sum_{i=1}^{l_x}}\left(1-{x}_i\right)$ the number of unmethylated CpG cytosines in *x*.

The sequence *x* may come from either normal or cancer cells, so the probability of observing *x* is:

$$ p(x)={\alpha}_1{p}_{x,1}+{\alpha}_2{p}_{x,2} $$

where *p*_*x*,*j*_ is the probability that sequence *x* is generated from the *j*-th component, *j* = 1,2. We assume that the methylation status of each cytosine in a genomic interval is independent, and *p*_*x*,*j*_ can be represented as

$$ {p}_{x, j}={\displaystyle \prod_{i=1}^{l_x\ }}\left({m}_j{x}_i+\left(1-{m}_j\right)\left(1-{x}_i\right)\right)={m_j}^{M_x}\cdot {\left(1-{m}_j\right)}^{U_x} $$

So the probability of observing the whole sequence set *X* from *I* is

$$ l(X)= p(X)={\displaystyle \prod_{x\in X}} p(x) $$

Given a set of bisulfite reads mapped to a genomic interval, the values of *m*_1_, *m*_2_, and *α*_1_ can be estimated by maximizing the log likelihood function

$$ \tilde{\varTheta}= \arg \underset{\varTheta}{ \max } l(X)= \arg \underset{\varTheta}{ \max }{\displaystyle \prod_{x\in X}} p(x)= \arg \underset{\varTheta}{ \max }{\displaystyle \sum_{x\in X}} \log p(x) $$

The optimization problem can be solved by the typical Expectation-Maximization (EM) algorithm by introducing a latent random variable z_*x*_ indicating the membership of sequence *x*:

$$ {z}_x=\left\{\begin{array}{c}\hfill 1,\mathrm{if}\; x\in \mathrm{minorcomponent};\hfill \\ {}\hfill 2,\mathrm{if}\; x\in \mathrm{majorcomponent}.\hfill \end{array}\right. $$

Let *Q*_*x*_(*j*) be the probability of *z*_*x*_ = *j*, then the log-likelihood function can be rewritten as

$$ l(X)={\displaystyle \sum_{x\in X}} \log p(x)={\displaystyle \sum_{x\in X}} \log {\displaystyle \sum_{j=1}^2}{Q}_x(j)\frac{\alpha_j{p}_{x, j}}{Q_x(j)} $$

According to the EM algorithm, if *Q*_*x*_(*j*) is estimated by the posterior probability of *z*_*x*_ given *x* and a pre-defined parameter setting *Θ*, that is,

$$ {Q}_x(j)= p\left({z}_x= j\Big| x;\varTheta \right)=\frac{\alpha_j{p}_{x, j}}{\alpha_1{p}_{x,1}+{\alpha}_2{p}_{x,2}} $$

Then by Jensen’s inequality, the above log-likelihood function can be estimated by

$$ \begin{array}{l} l(X)={\displaystyle \sum_{x\in X}} \log p(x)={\displaystyle \sum_{x\in X}} \log {\displaystyle \sum_{j=1}^2}{Q}_x(j)\frac{\alpha_j{p}_{x, j}}{Q_x(j)}\\ {}\ge {\displaystyle \sum_{x\in X}}{\displaystyle \sum_{j=1}^2}{Q}_x(j) \log \frac{\alpha_j{p}_{x, j}}{Q_x(j)}\triangleq J\left( X; Q\right)\end{array} $$

So,

$$ \begin{array}{l} J\left( X; Q\right)={\displaystyle \sum_{x\in X}}{\displaystyle \sum_{j=1,2}}{Q}_x(j)\left( \log {\alpha}_j+ \log {p}_{x, j}- \log {Q}_x(j)\right)\\ {}={\displaystyle \sum_{x\in X}}{\displaystyle \sum_{j=1,2}}{Q}_x(j)\left({M}_x \log {m}_j+{U}_x \log \left(1-{m}_j\right)\right)+{\displaystyle \sum_{x\in X}}{\displaystyle \sum_{j=1,2}}{Q}_x(j)\left( l\mathrm{og}{\alpha}_j- \log {Q}_x(j)\right)\end{array} $$

Setting $\frac{\partial J\left( X; Q\right)}{\partial {m}_j}=0$ and $\frac{\partial J\left( X; Q\right)}{\partial {\alpha}_1}=0$, we have

$$ \begin{array}{l}{\displaystyle \sum_{x\in X}}{Q}_x(j)\left(\frac{M_x}{m_j}-\frac{U_x}{\left(1-{m}_j\right)}\right)=0\\ {}{\displaystyle \sum_{x\in X}}{Q}_x(j)\frac{M_x-{l}_x{m}_j}{m_j\left(1-{m}_j\right)}=0\\ {}{m}_j=\frac{{\displaystyle {\sum}_{x\in X}}{Q}_x(j){M}_x}{{\displaystyle {\sum}_{x\in X}}{Q}_x(j){l}_x}\end{array} $$

and

$$ \begin{array}{l}{\displaystyle \sum_{x\in X}}{Q}_x(1)\frac{1}{\alpha_1}-{\displaystyle \sum_{x\in X}}{Q}_x(2)\frac{1}{1-{\alpha}_1}=0\\ {}{\displaystyle \sum_{x\in X}}\frac{Q_x(1)-{\alpha}_1{Q}_x(1)-{\alpha}_1{Q}_x(2)}{\alpha_1\left(1-{\alpha}_1\right)}=0\\ {}{\displaystyle \sum_{x\in X}}\frac{Q_x(1)-{\alpha}_1}{\alpha_1\left(1-{\alpha}_1\right)}=0\\ {}{\alpha}_1=\frac{{\displaystyle {\sum}_x}{Q}_x(1)}{\left| X\right|}\end{array} $$

So the final EM algorithm can be formulated as

(E-step): for each *x*

$$ {Q}_x(j):= p\left({z}_x= j\Big| x;\varTheta \right)=\frac{\alpha_j{p}_{x, j}}{\alpha_1{p}_{x,1}+{\alpha}_2{p}_{x,2}} $$

(M-step):

$$ \left\{\begin{array}{c}\hfill {m}_j=\frac{{\displaystyle {\sum}_{x\in X}}{Q}_x(j){M}_x}{{\displaystyle {\sum}_{x\in X}}{Q}_x(j){l}_x},\  j=1,2\hfill \\ {}\hfill {\alpha}_1=\frac{{\displaystyle {\sum}_x}{Q}_x(1)}{\left| X\right|}\hfill \end{array}\right. $$

Intuitively, the EM algorithm starts with a random guess of the model parameters *Θ* = (*m*_1_, *m*_2_, *α*_1_). In the E step, the algorithm computes the membership probability *Q*_*x*_(*j*) for each binary sequence given the current estimation of *Θ*. In the M step, *Θ* is re-estimated based on the membership probabilities *Q*_*x*_(*j*). By repeating the E steps and M steps recursively, the EM algorithm is proven to converge to a local maximum of log likelihood function [53].

### Determination of the mixing ratio

For most genomic bins, their methylation levels in tumor and its normal cells are roughly consistent. These bins are considered to be ‘non-informative’ in our estimation because any choice of *α*_1_ would lead to the same value of the likelihood function. Even if the bin is ‘informative’ (or has different methylation levels between normal and tumor cells), it is difficult to estimate the real mixing ratio precisely just from one bin due to the random noise and the insufficient read coverage. Known that all informative bins share approximately the same mixing ratio, we use the following strategy: we identify the informative bins through a bootstrap strategy, estimate the mixing ratio of each informative bin individually, and ‘vote’ for the real mixing ratio by combining the results from all those bins.

A bin is informative to determine the mixing ratio if it has enough read coverage and all reads in this bin are homozygous (that is, all the CpG dinucleotides in one read are methylated or non-methylated). So it is expected to get very reliable estimation even from partial data. We adopt the following strategies to search informative bins. First, we constrain our informative bin search on the CpG islands and nearby regions because these regions have high CpG density and highly variable methylation level compared to the normal cells [14]. Second, if a bin has no methylation difference between normal and tumor cells, the predicted mixing ratio will be randomly distributed between 0 and 1 due to the random initialization of parameters. As a result, we further select bins based on the variation of the parameter estimations from random sampling. We sampled all reads from a bin with replacement 50 times, and optimize the parameters based on the selected reads using the EM algorithm. We calculate the standard deviations of the three parameters across sampling, and bins whose parameters have lower standard derivations are selected. According to the simulation results, we found that the standard deviation of *m*_1_ is significantly higher than the other two parameters, and thus is adopted to rank all bins.

Copy number variations (CNVs) are frequently detected in tumor cells and may confound the results of estimating the mixing ratio. Theoretically, the amplified bins are more prone to be selected as informative bins due to their deeper read coverage compared to the normal bins, and the estimation results from these bins may be inaccurate due to the elevated read counts from the tumor component. So we collected genomic regions that confer frequent amplification or deletion at different types of cancer from TCGA [54,55] and discard informative bins located in these regions. In addition, in order to alleviate the problem of novel CNVs for a specific sample or new cancer types not covered by TCGA, we only keep up to one informative bin for each GpG Island.

After selecting informative bins and removing the effect of CNVs, for each sliding bin of length 300, we computed the informative divergence based on all reads mapped to it, and ranked all bins by the value of informative divergence. Bins with the smallest informative divergence are selected to determine the predicted mixing ratio. The informative divergence cutoff to determine the number of informative bins needs to be small enough to ensure the stability and large enough to include a sufficient number of informative bins for a reliable estimation. In our model, we selected the top 500 bins with standard deviation of *m*_1_ less than 0.1. However, if we could not get enough informative bins (less than 500) because the sequence depth and read length is not enough, or the normal cell contamination is less than 5%, then our program (MethylPurify) will stop and report an error.

### Finding differentially methylated regions

With the predicted mixing ratio in mind, we next estimate the other two free parameters in our EM algorithm for each sliding bin across the genome. The computation is very similar as the previous three-parameter estimation model, except for a known *α*_1_, we thus omit the detail deduction in this part. A bin is called ‘differentially methylated’ if the predicted methylation ratios between normal and tumor cells are larger than a given threshold (0.5 in our simulation experiments). Finally, adjacent differentially methylated bins are merged together to get the differentially methylated regions.

The likelihood function may not be unimodal and may contain up to two local maxima (see Additional file 7: Figure S7 as an example). To handle this situation, our algorithm starts with two different initial values of *m*_1_ and *m*_2_ (that is, *m*_1_ = 0.8, *m*_2_ = 0.2 and *m*_1_ = 0.2, *m*_2_ = 0.8), and the convergence point with higher likelihood probability is selected as the final prediction.

### Data access

The BS-seq data used in this manuscript have been deposited in the NCBI Gene Expression Omnibus (GEO) under accession number GSE56712.

### Availability

MethylPurify is available open source at https://pypi.python.org/pypi/MethylPurify.

## Additional files

Additional file 1: Figure S1. Distribution of informative bins over chromosomes. Each red bar shows an informative bin inferred from the cell line mixture of HCC (0.7) and HMEC (0.3). Informative bins are defined as the top 500 qualifying bins (300 bp in length with > =10X coverage and > =10 CpG) that with smallest parameter variance by EM bootstrap. Additional file 2: Figure S2. DMB prediction at different read coverage. Bisulfite reads from two cell lines are randomly sampled to make sure the mixing ratio of HCC and HMEC (HCC:0.7, HMEC:0.3) at different read coverage (from 10-fold to 40-fold). Sensitivity and specificity are obtained by the direct comparison between the two cell lines as benchmark. Covered bins are qualifying bins in the genome with enough read coverage and CpG counts. Additional file 3: Figure S3. Distribution of Methylation levels of true positive **(a)** and false positive bins **(b)** in HMEC and HCC cell lines. The DMBs called by direct comparison between tumor and normal cell lines (difference >0.5) were treated as benchmark to evaluate the predicted DMBs identified by MethylPurify. In each subfigure, the white dot shows the median, the thick black bar represents the interquartile range, and the thin black bar represents 95% confidence intervals. Additional file 4: Figure S4. Overlap between predicted DMBs and true DMBs after removing the inconsistent bins in HCC. True DMBs (blue) are derived by the direct comparison between the two cell lines (difference >0.5); Predicted DMBs (red) are differentially methylated bins (difference > 0.5) identified by MethylPurify only from the simulated cell mixture (HCC:0.7, HMEC:0.3). The inconsistent bins were defined as bins that contain inconsistent reads (SD of observed reads methylation level >0.1) and were considered as heterogeneous regions. Additional file 5: Figure S5. Impact of allele-specific methylation (ASM) to mixing ratio prediction. **(a)** Suppose the mixing ratio of a tumor tissue is 0.4 (normal): 0.6 (tumor), and within a region the normal part is allele-specific methylated while the tumor part not. **(b)** MethylPurify is inclined towards separating reads into fully methylated and fully unmethylated populations, thus **(c)** predicting the mixing ratio to be 0.2 (unmethylated): 0.8 (methylated). Additional file 6: Figure S6. Mixing ratios and DMB prediction for two metastatic lung cancer samples. **(a)** Distribution of informative bins and the calculated minor components of two metastatic lung cancer samples. **(b)** DMBs in blue were inferred directly by comparing normal and tumor tissues, DMBs in red were predicted by MethlPurify from only tumor tissues, and DMBs in green were summarized from TCGA (bins that frequently show altered DNA methylation in lung cancers). Additional file 7: Figure S7. Contour plot of log likelihood function for a typical DMB. The log likelihood function of a bin is summed from all reads mapped to this bin by varying two free parameters *m*_1_ and *m*_2_ from 0 to 1. The red triangle shows a local maxima, while the red star is the global optimum.

## Abbreviations

HCC: HCC1954 breast cancer cell line
HMEC: Primary human mammary epithelial cells
RRBS: Reduced representation bisulfite sequencing
TCGA: The cancer genome Atlas
WGBS: Whole genome bisulfite sequencing
