# Supplementary figures and images for: MethylPurify: tumor purity deconvolution and differential methylation detection from single tumor DNA methylomes
Source: Genome Biol. 2014 Aug 7;15(7):419. doi: 10.1186/s13059-014-0419-x (PMC4165374; doi:10.1186/s13059-014-0419-x)

Distribution of informative bins over Chromosomes

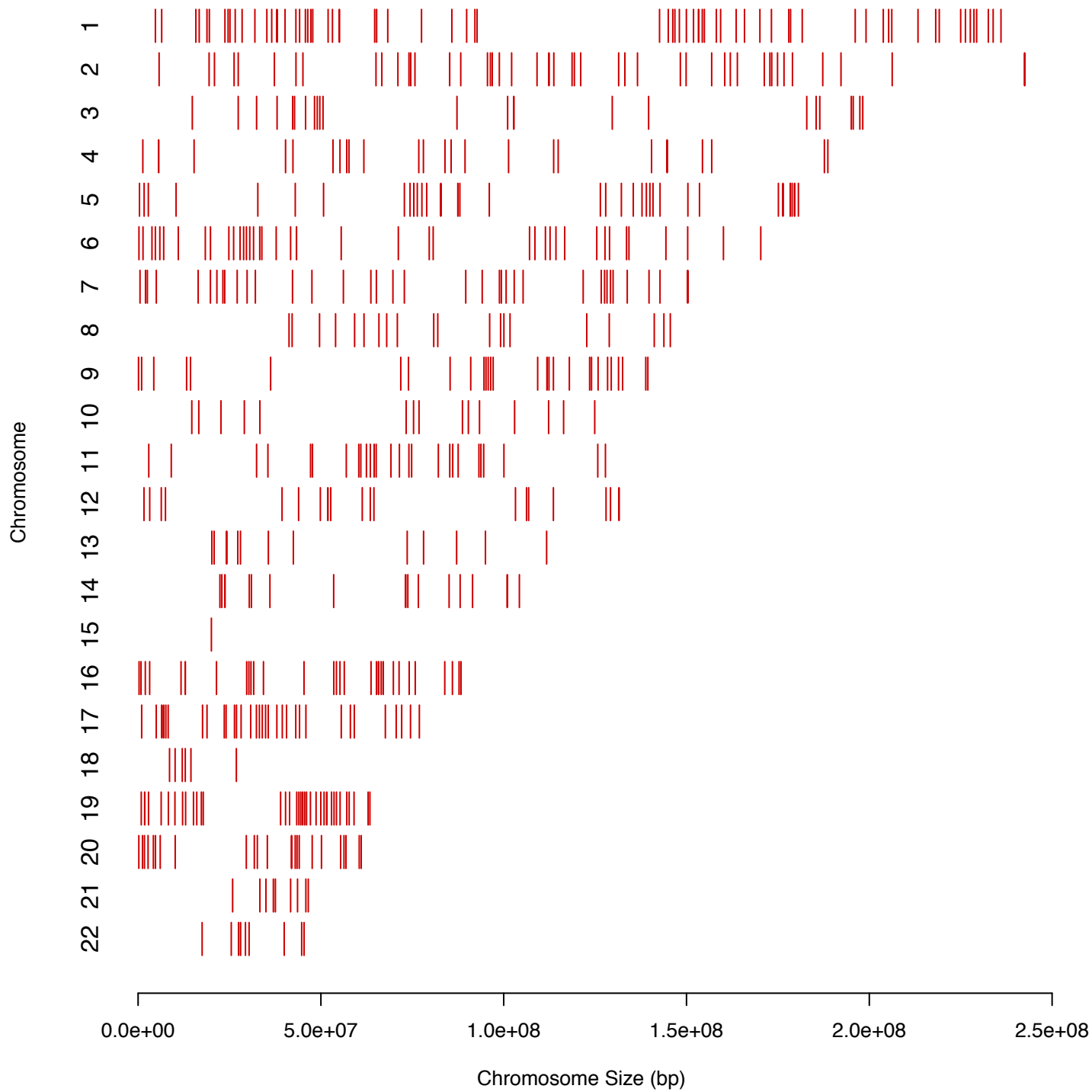

Supplement: Additional file 1: Figure S1. — Distribution of informative bins over chromosomes. Each red bar shows an informative bin inferred from the cell line mixture of HCC (0.7) and HMEC (0.3). Informative bins are defined as the top 500 qualifying bins (300 bp in length with > =10X coverage and > =10 CpG) that with smallest parameter variance by EM bootstrap. [file 13059_2014_419_MOESM1_ESM.pdf]

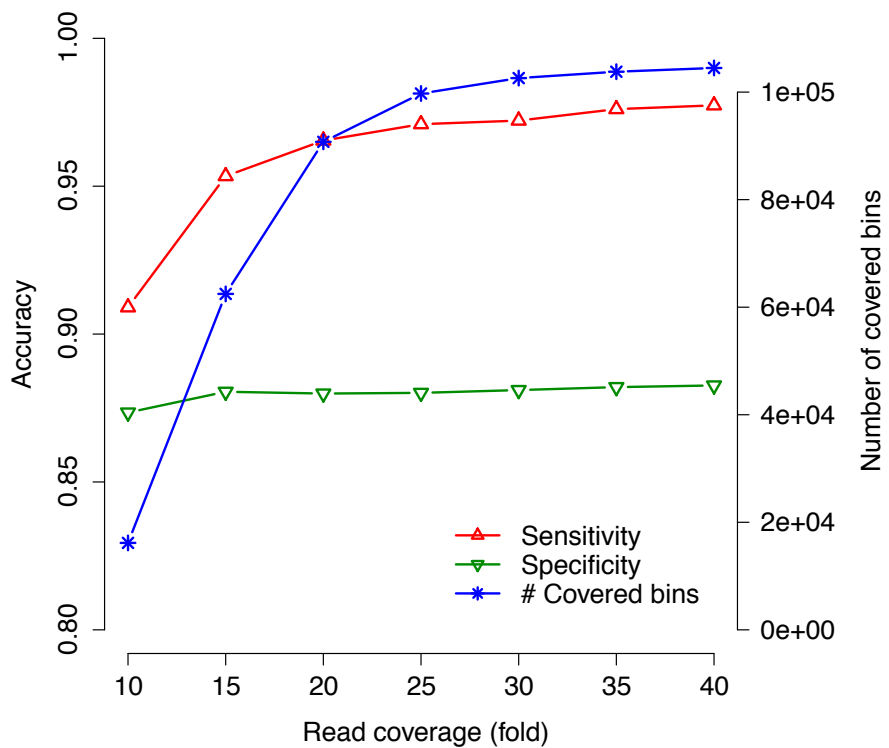

Supplement: Additional file 2: Figure S2. — DMB prediction at different read coverage. Bisulfite reads from two cell lines are randomly sampled to make sure the mixing ratio of HCC and HMEC (HCC:0.7, HMEC:0.3) at different read coverage (from 10-fold to 40-fold). Sensitivity and specificity are obtained by the direct comparison between the two cell lines as benchmark. Covered bins are qualifying bins in the genome with enough read coverage and CpG counts. [file 13059_2014_419_MOESM2_ESM.pdf]

**a**

True positive bins

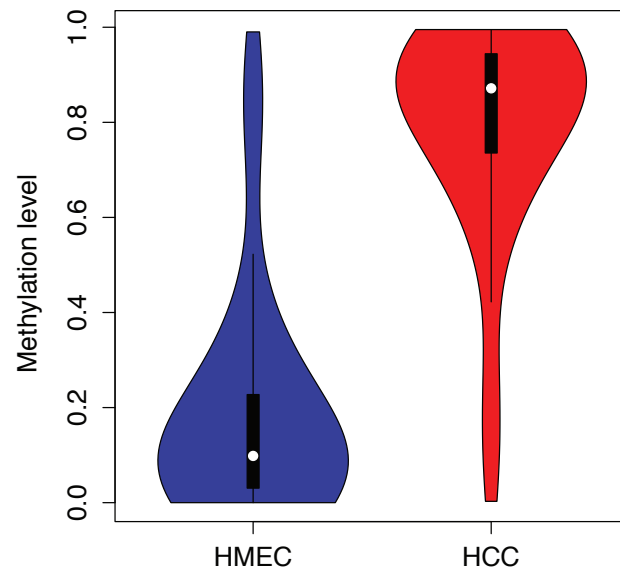**b**

False positive bins

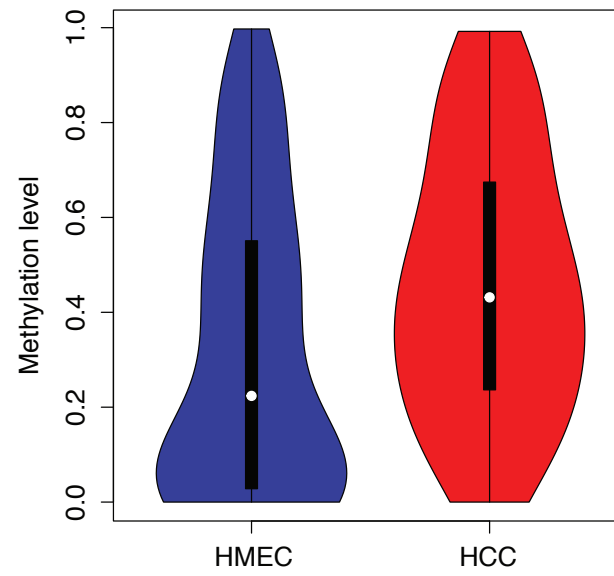

Supplement: Additional file 3: Figure S3. — Distribution of Methylation levels of true positive (a) and false positive bins (b) in HMEC and HCC cell lines. The DMBs called by direct comparison between tumor and normal cell lines (difference >0.5) were treated as benchmark to evaluate the predicted DMBs identified by MethylPurify. In each subfigure, the white dot shows the median, the thick black bar represents the interquartile range, and the thin black bar represents 95% confidence intervals. [file 13059_2014_419_MOESM3_ESM.pdf]

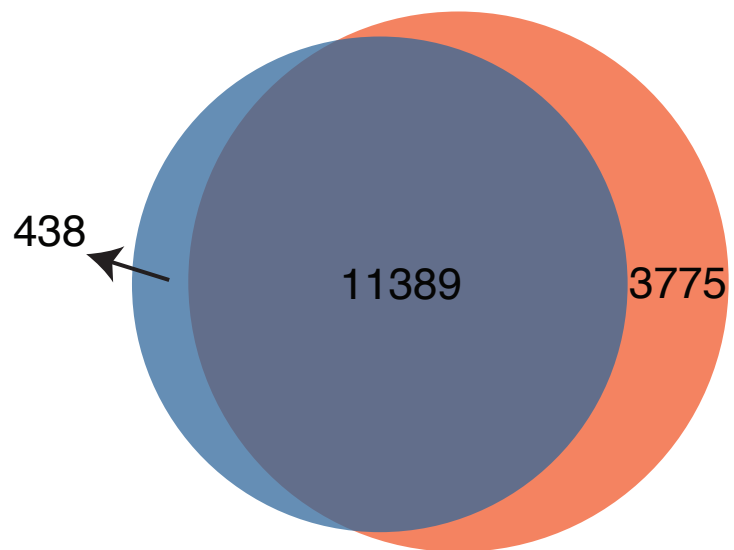

- True DMBs
- Predicted DMBs

Supplement: Additional file 4: Figure S4. — Overlap between predicted DMBs and true DMBs after removing the inconsistent bins in HCC. True DMBs (blue) are derived by the direct comparison between the two cell lines (difference >0.5); Predicted DMBs (red) are differentially methylated bins (difference > 0.5) identified by MethylPurify only from the simulated cell mixture (HCC:0.7, HMEC:0.3). The inconsistent bins were defined as bins that contain inconsistent reads (SD of observed reads methylation level >0.1) and were considered as heterogeneous regions. [file 13059_2014_419_MOESM4_ESM.pdf]

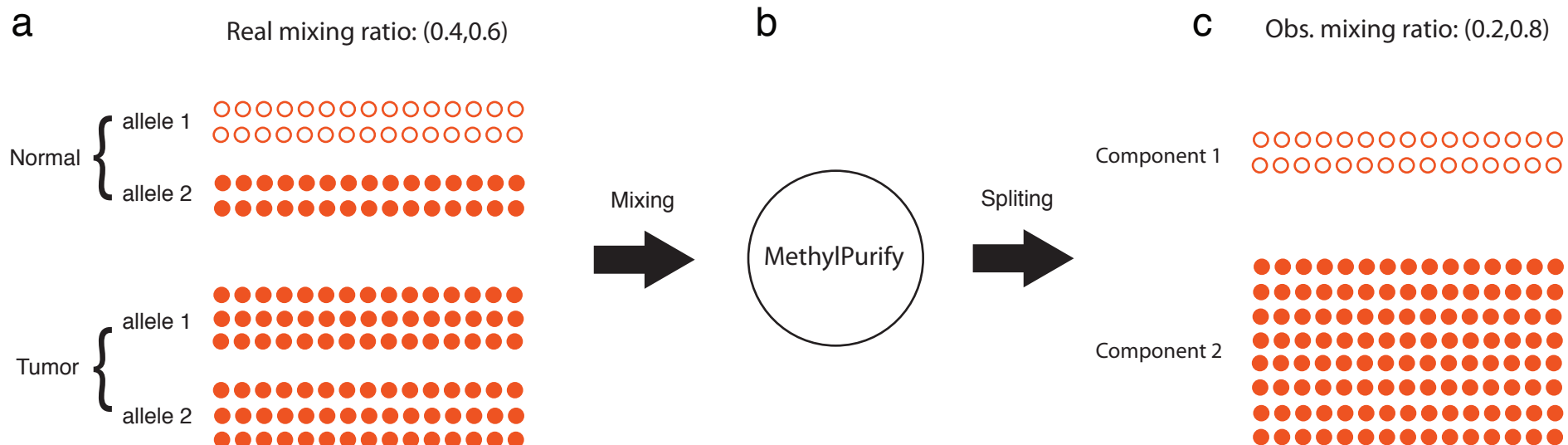

Supplement: Additional file 5: Figure S5. — Impact of allele-specific methylation (ASM) to mixing ratio prediction. (a) Suppose the mixing ratio of a tumor tissue is 0.4 (normal): 0.6 (tumor), and within a region the normal part is allele-specific methylated while the tumor part not. (b) MethylPurify is inclined towards separating reads into fully methylated and fully unmethylated populations, thus (c) predicting the mixing ratio to be 0.2 (unmethylated): 0.8 (methylated). [file 13059_2014_419_MOESM5_ESM.pdf]

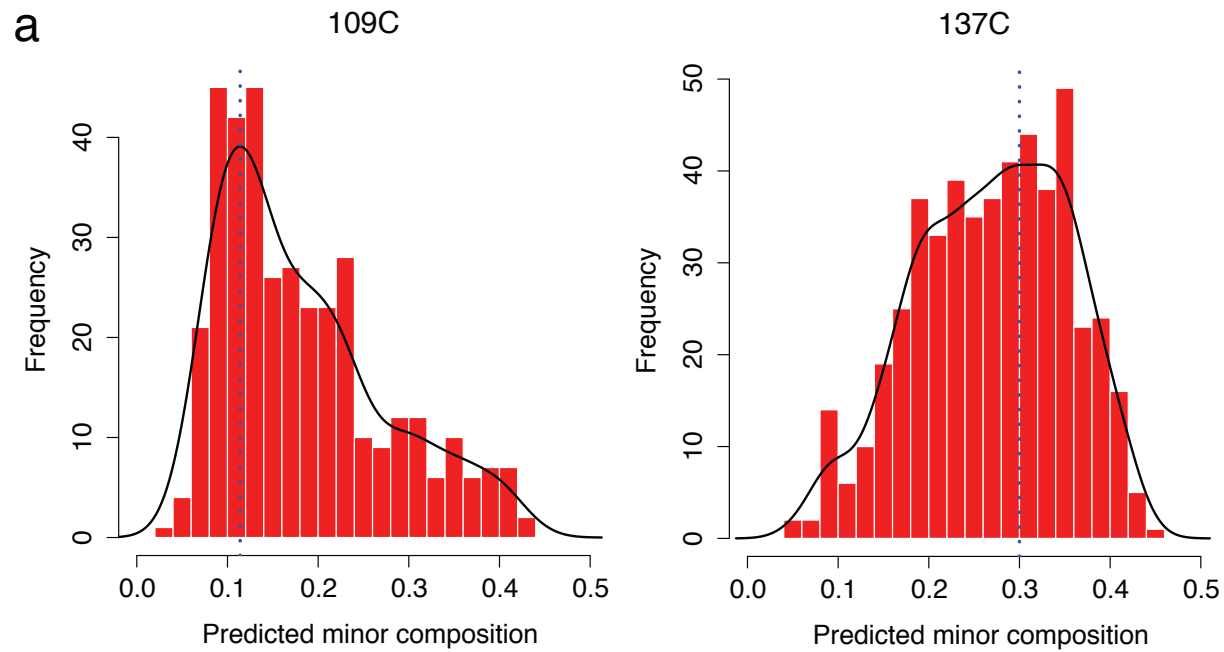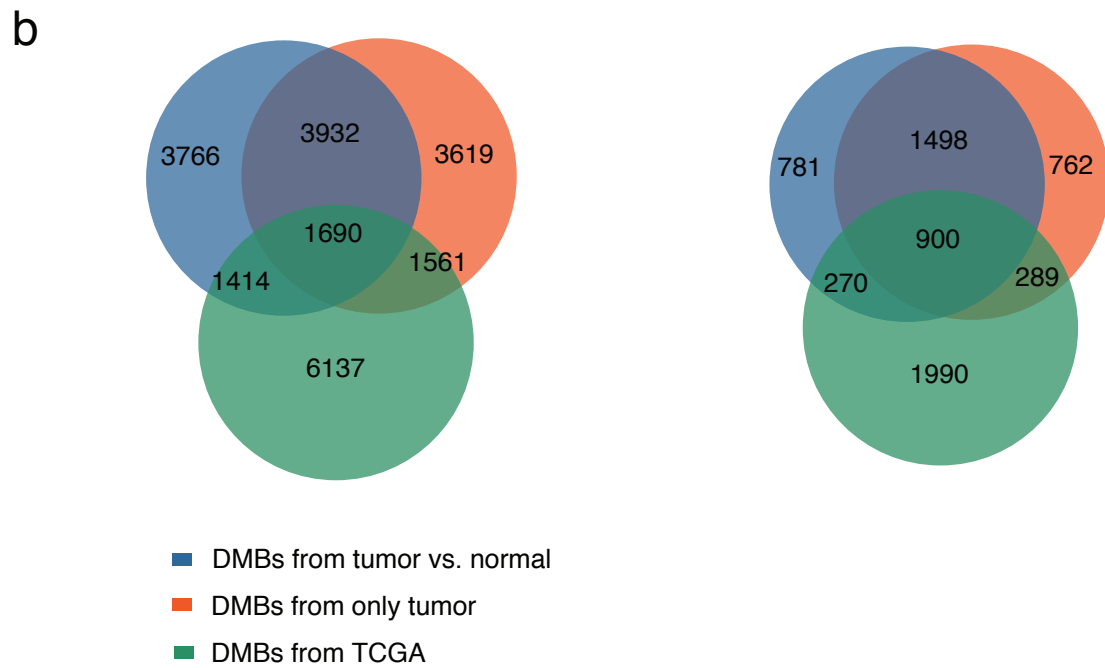

Supplement: Additional file 6: Figure S6. — Mixing ratios and DMB prediction for two metastatic lung cancer samples. (a) Distribution of informative bins and the calculated minor components of two metastatic lung cancer samples. (b) DMBs in blue were inferred directly by comparing normal and tumor tissues, DMBs in red were predicted by MethlPurify from only tumor tissues, and DMBs in green were summarized from TCGA (bins that frequently show altered DNA methylation in lung cancers). [file 13059_2014_419_MOESM6_ESM.pdf]

## Contour plot of log likelihood function

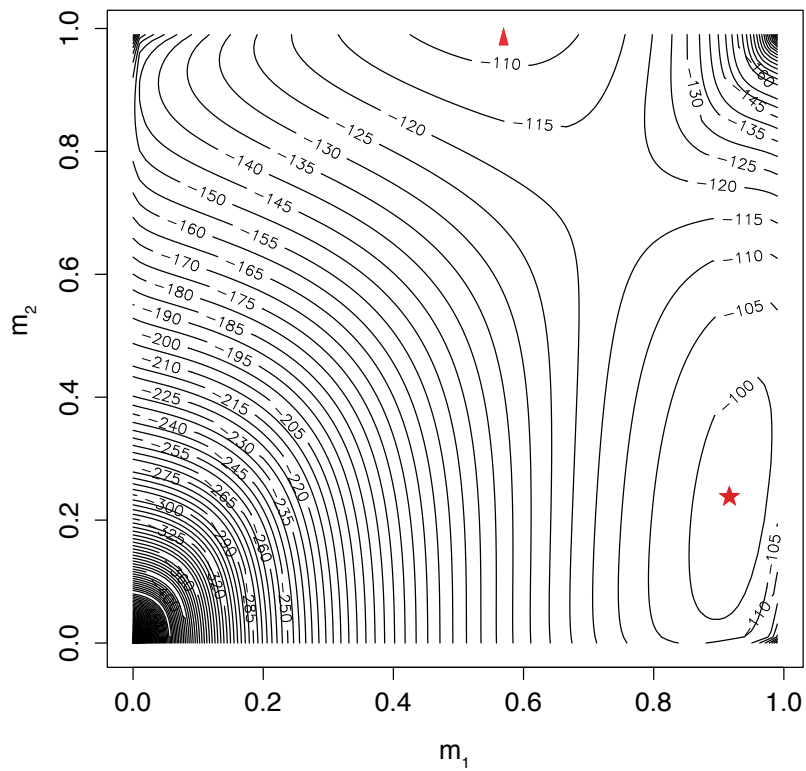

Supplement: Additional file 7: Figure S7. — Contour plot of log likelihood function for a typical DMB. The log likelihood function of a bin is summed from all reads mapped to this bin by varying two free parameters m 1 and m 2 from 0 to 1. The red triangle shows a local maxima, while the red star is the global optimum. [file 13059_2014_419_MOESM7_ESM.pdf]
